# Therapeutics and Materia Medica

**Published:** 1857-05

**Authors:** 


### THERAPEUTICS AND MATERIA MEDICA.

1. STRYCHNIA: ITS USES AND ABUSES.—"This powerful alkali has figured very prominently of late before the public; and has, in certain instances, been handled, medically, in a manner somewhat remarkable. It is certainly not from any lack of caution as to its use by writers upon Pharmacy and Therapeutics that its powers have been at times very strikingly and dangerously manifested; but it is rather owing to its reckless employment, or to an over-zeal in eliciting its effects, that accidents under medical management have happened. Those in the habit of prescribing it, if well instructed, know that it is second only to prussic acid in energy, when given in sufficient quantity to affect the system as a poison. A girl, 13 years old, died in about one hour from taking, by mistake, three-fourths of a grain divided into three pills; and it has even been asserted that merely inhaling a little of it has proved fatal. Moreover, administered remedially, it sometimes has had an evidently cumulative action, and its effects are very likely to break out suddenly and uncontrollably, unless the greatest care is taken not to give too large doses, continuously. Yet there are instances where, from having long given it ineffectually, the practitioner has become impatient and added, very slightly it may be, to the usual dose, with the result of throwing his patient into strong convulsions. At other times the increase of the dose has even been more rash. Certainly this is an *abuse* of strychnia.

"We have lately heard of employing strychnia in some cases of *insanity*. We do not deny that there may be instances where it is demanded—as perhaps in certain concomitant paralytic states; but we are not cognizant of any special action that is predicated of this medicine likely to benefit the mental aberration. In a case of furious mania recently for a short time under our observation, we learned that *strychnia* had been administered on the outbreak of the affection. We are aware that it has been recommended in certain cases of maniacal aberration—but, as we suppose, in such as exhibited the moping melancholy form, and in hypochondriacal states. We fail to see the indication for its employment in violent, active mania, in young, vigorous persons. If we mistake not, there have lately been reports of similar treatment in analogous cases; if our distrust be only *ignorance*, we beg to be enlightened upon the point. Unless we are thus informed, we put this down as another abuse of strychnia.

"It is needless to refer to the frightful and cold-blooded murders, the detailed circumstances of which have made communities tremble. In these cases, the abuses of strychnia has had its *uses*, in that it has given to the world the elaborate chemical reports and investigations required by the legal necessities of the case. These will stand as invaluable evidence, and will be always looked upon as mines of information. In connection with this part of our subject, we consider it an *abuse* of strychnia, as of *any* subtle and potent poison, to have it so easily procured. Druggists should not be allowed to vend this medicine, any more than arsenic, opium, prussic acid, &c., to all applicants indiscriminately. Might not much of this abuse be done away by refusing the sale to all who do not present a physician's prescription or order? We are aware that much has been written and said upon this point, and also that nothing, of consequence, has been done. Often these deadly articles are as heedlessly sold as the most simple remedies. The small pecuniary gain to the apothecary, levies a large debt of responsibility against him.

"The legitimate *uses* of strychnia are well known. A powerful excitant of the nervous system, without any specific action on the brain, it has been long acknowledged to be a very valuable remedy in certain paralytic conditions. Combined in minute doses with purgatives, it hastens and increases their action; and it has thus been advantageously employed in some cases of amenorrhœa, or of suspended menstruation. We can testify to good service done by it in this way. As a tonic, *brucia*, the other component alkaloid of nux vomica, has been found perhaps more useful than strychnia. The latter is often prescribed in dyspeptic states, such as are accompanied by pyrosis and gastrodynia. Testimony is strongly favorable to its curative effects in spasmodic asthma. Externally, its employment for amaurotic troubles has been extensive.

"To recur once more to the abuses of strychnia, or, which amounts to the same thing, of the nux vomica, we cannot refrain from alluding to one which, in view of the strength and unmanageable nature of the agent, should be represented to the too credulous public in the way of a caution. There are those who, by the necessity of their position and avocations, cannot have that knowledge of it, and familiarity with this and other giants of the Materia Medica, which fit them for advising or regulating their use. Still, very many, in every community, are willing to take from such unskilled persons, compounds containing unknown amounts of strychnia, &c. &c. Thus we have *soidisant* or retired clergymen advertising that they will furnish a prescription for a preparation containing the active principle of St. Ignatius's bean, and the direction for using the same. All such tamperers with human strength and life are accountable to a higher tribunal than any earthly one, and those who aid and abet them must bear them company thither. It being quite sure that the adoption of these quack remedies by the people, only brings the honest physician more patients, we shall not be accused of covetousness in protesting against them. We do not aspire to coerce people, even by argument and the exposition of the bold and unwarrantable assumption that seeks to medicate—or rather to poison them—they are free agents, but certainly in no other affairs do they act so inadvisedly or expose precious interests so recklessly as in the care (as they understand it) of their health.

"The proper uses of strychnia, as of all medicinal agents, are only thoroughly known by the educated physician. Why does any one desire—or dare—having the manifest peril in view which its improper employment implies—to intrust its administration to the unfamiliar—the adventurer—or still worse, if possible, to their own judgment?

"And we commend to legislative consideration the dangers constantly attendant upon the unrestricted sale of medicinal articles, a fractional part of a grain of which sometimes takes life more quickly than the knife or the bullet. The facility of procuring such materials arms the unprofessional murderer quite as surely, if less covertly, than it does a PALMER."—*Boston Med. and Surg. Journ.* Feb. 26, 1857.

2. ARSENICAL POISONING.—"Dr. Hinds, of Birmingham, has lately called attention to a method of accidental arsenical poison, which should be generally known, and from which he was himself a sufferer. He chanced to select, for the adornment of his study, a particularly bright-tinted wall-paper, the pattern of which was confined to two shades of green. About two days after it had been applied, he first used the room in the evening, sitting there, and reading by a gas-light. Whilst thus engaged, he was seized with a severe depression, nausea, abdominal pain, and prostration. The same chain of symptoms ensued on every subsequent evening when he occupied the room. This lead to an inquiry into the cause. He scraped off a little of the bright coloring-matter from his pretty green paper, and, by sublimation, produced abundant crystals of arsenious acid. The paper was colored with arsenite of copper (Scheele's green). The use of this pigment to color wall-papers has already proved injurious in previous cases. In one, a child sucked some strips of paper thus colored, and narrowly escaped with life. (Ed. *Monthly Journal,* 1851.) Dr. Hinds remarks, that the presence of the arsenical pigment may be recognized by its brilliant and beautiful hue, and by a little running of the color at the edges of the pattern, as though it did not take freely to the paper."—*Lancet*, Feb. 21, 1857.

3. ABSORPTION OF MEDICINAL SUBSTANCES INTRODUCED AS ENEMATA INTO THE LARGE INTESTINE.—M. Briquet read a memoir on this subject, before the Academy of Medicine, on the 30th of December last, principally in reference to quinia and its salts. The author draws the following conclusions:

"1. The fluid of which the enemata consists may readily enough be carried as far as the cœcum, and consequently be applied to an extensive absorbent surface.

"2. The mucous membrane of the large intestine, and the liquids which bathe its surface, exert no chemical influence over the substances introduced into this cavity, in which nothing is absorbed but what was previously in solution.

"3. When we administer by injection, per anum, salts of quinine, in solution, in a dose less than fifteen grains, a little more than a third of this quantity has disappeared, and has consequently been absorbed.

"4. When doses larger than fifteen grains are administered, they are badly received, and not more than a fifth, or even a sixth of the quantity is absorbed.

"5. At whatever dose we give the sulphate of quinine [by injection?] cerebral symptoms are, in common, produced only very slowly, and in minor degree.

"6. No traces of absorption are perceived before an hour has elapsed after the administration of the enema, and at this time but an inconsiderable quantity has been thus disposed of.

"7. The duration of the absorbing process is, in general, rather short, and seldom extends beyond two or three days at most.

"8. The absorption of the alkaloids of cinchona is not sensibly affected by various degrees of dilution, within, be it understood, certain limits, by the greater or less viscosity of the liquid, nor, finally, by the addition of the salts of morphia.

"9. Young people absorb better than adults; old persons of either sex absorb very imperfectly.

"10. The alkaloids of cinchona, administered by enema, in doses less than fifteen grains, may produce by this means all the good effects to be expected from the alkaloids given in small doses by the mouth, and may very well be substituted for the latter.

"11. The case is different with large doses; which are never absorbed in sufficient quantity to produce energetic stupefying effects [quininism?].

"12. In general, larger doses than thirty grains of sulphate of quinine are not borne by the large intestine.

"These conclusions are applicable in a greater or less degree to the various substances employed as enemata."—*Rev. de Therap. Med. Chirurg.*, Jan. 15.

We may expect from the report of a Committee or Commission, appointed by the Academy of Medicine, for the purpose of an analysis of the conclusions reached by M. Briquet, which, with apparent mathematical precision, seem to be deficient in their application to clinical medicine.

4. NITRATE OF POTASH IN LARGE DOSES IN ASCITES WITH ANASARCA.—We read in the Rev. de Ther. Med. Chir. (Jan. 15th, 1857), an interesting account, translated from the Chronica de las Hospitales, of a case of a man who, in eighteen days, was entirely relieved of ascites and anasarca by the use of large doses of the above remedy. On the first day of treatment, which was, as near as we can ascertain, about six weeks from the coming on of the dropsy, and upwards of seven weeks from the beginning of an attack of remittent fever which preceded the dropsy, the new medical attendant, M. Angulo, directed for his patient two drachms of nitrate of potash dissolved in a saccharo-mucilaginous vehicle, which was to be taken in a period of thirty hours. On the first day of treatment there was a slight increase of the urinary secretion; the stomach bore the medicine very well. On the following day the nitre was increased to rather more than three drachms. The patient had some alvine evacuations; the urine was more abundant than on the preceding day. On the third day the patient was much better. At his request the same medicine was given, the doses of which were successively raised until, on the eleventh day of treatment, they had reached eleven drachms two scruples, or nearly an ounce and a half in the twenty-four hours. On the twelfth day the body had resumed its customary size, after an excessive urinary discharge, which had supervened on the fifth, sixth, and seventh days.

In proportion as the serous effusion was absorbed, the appetite and strength were restored, and dating from the seventh day's administration of the nitrate of potash, the patient was able to walk about his room. At the time he came under the charge of M. Angulo, this man was unable to leave his bed, and so great was the anasarca and swelling of the eyelids that the globe of the eye was no longer visible.

The regimen consisted of preserved fruits, baked apples, panada, vermicelli soup, a little good wine, and at a later period a more abundant alimentation. After the twelfth day the dose of the nitrate of potash was diminished, and on the nineteenth its use was abandoned. On the 8th of January, 1856, or seven weeks and two days from the beginning of the nitre treatment, the man was so entirely restored to health, that he resumed his business on the road in trading in wine, which he transported by mules.

[The above narrated case is the more instructive, as nitre was the only remedy employed; and taking into consideration the state of the patient before it was prescribed, and the prompt change produced by this medicine, and the complete removal of the disease under a continuance of its use, we cannot resist the inference that it was the undoubted and sole curative agent. We may draw also another inference from a knowledge of this case, viz., that much yet remains for us to learn respecting the therapeutic nature of many medicinal substances with which we think we have a familiar and intimate acquaintance; and that, as our French contemporary justly observes, the art of handling them, if we may use the expression, will be richer in results than any physico-chemical speculations that may be indulged in.]

5. CHLORATE OF POTASH IN MEMBRANOUS FORMATIONS AND ULCERATIONS OF THE MOUTH AND THROAT.—In three successive numbers (from November 1st to

December 15th), of the *Revue de Therapeutique Medico-Chirurgicale*, M. Isambert has given some interesting clinical details on the therapeutic value of chlorate of potash in various forms of stomatitis and angina, the more prominent of which we shall lay before our readers. In the ulcero-membranous stomatitis of Rilliet and Barthez, the fibrinous or ulcerous stomatitis of other writers, chlorate of potash would seem to be entitled to the preference over all other remedies. West has spoken of it as meriting the title of specific. Under its use there is an astonishing amelioration of the disease as early as the second or third day, and from the seventh to the tenth day the cure is complete. This writer directs three grains in a sweetened solution every four hours, to a child three years old; and five grains to one eight or nine years of age. It is admissible in all the stages of the disease. When constipation is present a purgative should precede the use of the chlorate. Equally gratifying results were announced, by M. Blache, of his experience in the Hospital for Children. Under the use of the chlorate of potash, the children, five in number, affected with ulcero-membranous stomatitis, were cured in five or six days, and without their being subjected to a relapse; whereas, in six cases which were treated by cauterization with muriatic acid and chloride of lime, the mean duration of the disease was twenty days. To the same purport are the cases, two in number, furnished by M. Barthez, and in a still more startling degree the twenty-one cases treated by M. Bergeron, in the Military Hospital at Roule.

M. Isambert makes some useful observations in confounding the experience of others in this matter, and at the same time offering some new suggestions to enable us to meet certain contingencies. He recommends a continuance in the use of the chlorate some time after the detachment of the false membrane, in order to prevent a relapse; and he regards preliminary cauterizations as uncalled for; while, at the same time, he thinks this salt powerless in inter-alveolar pyorrhœa. Often the ulceration is diminished and even cured, at the same time that the false membrane comes away. Even where the excoriated surface is seen after the detachment of the membrane, it is soon cured. In other cases again, it must be admitted that this condition of the part remains stationary, and the chlorate of potash produces no effect.

In aphthæ, or a vesico-ulcerous state of the buccal mucous membrane, seated in the muciparous follicles, chlorate of potash has been used with advantage. M. Legroux has derived good effects from this salt in the white thrush (muguet), the pultaceous or curdy stomatitis. In scurvy, recent experience is confirmatory of the opinion advanced several years ago, of the efficacy of chlorate of potash.

In angina maligna, in fibrinous angina, the chlorate of potash begins to figure as an important remedy. A pathological remark of some interest may be made here. It is, that all the morbid depositions of a membranous character on the mucous membrane of the mouth, pharynx, larynx, and trachea, from the elastic ribbon-like membrane of croup to the pultaceous coat in the mouth, and over the tonsils, of the least cohesive nature, and including that in angina maligna, and scarlatina, and in fibrinous stomatitis, exhibited under the microscope, to the observation of M. Robin, an identical composition; that is to say, that they consisted of almost pure fibrin, often a little mixed with epithelial remains, and some globules of pus and blood. The difference of cohesion, so much insisted on, depends only on a difference in molecular grouping, and in the smallest pultaceous shred the microscope shows the fibrillary structure of fibrin. This sameness in the product of exudation in the diseases just named, does not, by any means, imply a sameness in their other pathological features, and certainly not in the therapeutical means by which they are combated. In the matter of prognosis it has been observed, that whenever, in membranous angina, the cervical glands are much affected by engorgement and swelling, we must apprehend an alarming condition of the patient, if not a fatal termination of the disease.

Four cases of membranous angina in children are detailed by M. Isambert, in which cures were obtained by the use of the chlorate of potash alone. In four others its administration did not prevent a fatal result. As auxiliary means, it was observed that simple astringent gargles, or such detersive injections were preferable to caustics, which, in some cases, seemed, so far from accelerating to retard the curative action of the chlorate of potash. The utility of these latter is displayed after the false membrane comes away. Real adjuvants were found in the internal use of tonics, and especially in the preparations of cinchona.

6. Jelly of Iceland Moss and Cod-Liver Oil.—Professors Estor and Alquié have spoken favorably of a preparation made by M. Sauvan, of Montpellier, in thoracic affections. The following are the formula and mode of preparation of this new combination:

| | | |
|---|---|---|
| Jelly of Iceland Moss, . . . . . | 125 grammes | (32 drs). |
| Gelatine, . . . . . . . . | 5 " | (4 scruples). |
| Cyanhydrated Cod-Liver Oil (with two drops of essence of bitter almonds), . . . | 125 " | (32 drs.) |

Prepare the jelly of moss in the usual manner; melt the jelly, and pour it into the vessel into which it is to be kept. Add then the cod-liver oil; stir with a spatula until the mixture is homogeneous, and until the jelly begins to form.

This jelly is to be given in the same doses as the cod-liver oil, two or three tablespoonfuls a day. Professor Estor adds to the above formula fifteen drachms of the syrup of phellandrium.—*Bulletin de Thérapeutique.*

7. Mode of Administering Iodine in large Doses with entire Safety—M. Lasègue asserts, after numerous trials of the article, that the internal administration of tincture of iodine, in doses beyond those commonly given, is quite safe. In order, however, to avoid painful sensations, a kind of gastralgia, which is often induced by the use of this medicine, it ought to be taken only at meal-time; so that the gastric excitement produced at this time will not be productive either of pain or injury, but will rather favor digestion. With this precaution M. Lasègue has progressively increased the dose from 8 or 10 drops to four scruples, and a drachm and a half, during the meal; the patient taking as an excipient a little sweetened water, or, preferably, Spanish wine.

The physiological effects long since described by Lugol have been regularly noticed, as well as the therapeutical results, without there ever having occurred the slightest accident in evidence of a temporary intoxication (iodinism).—*Révue Thérap.*

8. TINCTURE OF IODINE FOR VOMITING IN PREGNANCY.—Dr. Eulenburg, of Coblentz, tells us, in the *Gazette Médicale Etrangère*, that this tincture, even in small doses, is one of the best agents for the relief of the vomiting, which so often distresses pregnant woman. He prescribes it very diluted, as follows: tincture of iodine, one part; rectified alcohol, four parts and a half; mix. He directs of this diluted tincture three drops in a little water. This remedy, in addition to its anti-emetic effect, calms the often accompanying cardialgia and gastralgia. He has not derived the same good effects, in these affections, from the iodide of potassium.

9. IODINE ENEMATA IN THE TENESMUS OF DYSENTERY.—In an epidemic dysentery, which prevailed in Wurtemberg, in 1854 and 1855, Doctor Palm was very successful in calming the tenesmus which so much tormented the sick, with an enema, consisting of iodine, 1½ grains; iodide of potassium, 15 grains, in an ounce of the vehicle. [Though not directed by Doctor Palm, we would recommend that the iodine and its salt be previously dissolved in a small quantity of water, and then mixed with mucilage of flaxseed or gum. Starch would of course be objectionable.]

The iodine injections, repeated once or twice daily, and continued for two or three days, were uniformly followed by feelings of great comfort, the more noticeable because other kinds of enemata, vapor bath, &c., were either inefficacious, or at the most, had only a palliative effect. The efficacy of iodine enemata was first pointed out by M. Eimer.—*Wurtemb. Corresp. Blatt.* 1856.

